# Histological effect of fluoride varnishes on teeth with caries in the white spot phase: An *in vitro* study

**DOI:** 10.4317/jced.62010

**Published:** 2025-04-01

**Authors:** Margot Margarita Gutiérrez-Ilave, Rocío del Pilar Ríos-León, Antonia Castro-Rodríguez, María Rosario Calixto-Cotos

**Affiliations:** 1 DDS. MSc., Professor of the Department of Preventive and Social Stomatology, Faculty of Dentistry, Universidad Nacional Mayor de San Marcos. Lima, Perú; 2 DDS., Master Student in Dentistry, Faculty of Dentistry, Universidad Nacional Mayor de San Marcos. Lima, Perú; 3 DDS. MSc., Professor of the Department of Preventive and Social Stomatology, Faculty of Dentistry, Universidad Nacional Mayor de San Marcos. Lima, Perú; 4 DDS. MSc., Professor of the Department of Analytical Chemistry. Faculty of chemistry and chemical engineering. Universidad Nacional Mayor de San Marcos. Lima, Perú

## Abstract

**Background:**

The aim of this study was to compare the histological remineralization effects of two fluoride varnishes on artificially produced caries lesions in young permanent teeth.

**Material and Methods:**

Twenty longitudinal sections of premolars with artificially produced white spot lesions and evaluated with a polarised light microscope. The sample consisted of 20 tooth fragments, which were divided and assigned to two groups: The first group with application of Clinpro TM White Varnish (20 tooth sections) and the second, with MI Varnish TM (20 tooth sections) subjected for 6 days to conditions similar to the oral cavity. The sections were then re-evaluated by calculating the lesion depth and remineralization area (Image J software). Student’ s t-test was used to analyse the data.

**Results:**

MI Varnish TM fluoride varnish achieved a greater remineralization area than Clinpro TM White Varnish (838042.6±140359.3 μm2 and 678313.8±137265.7μm2, respectively), with a statistically significant difference (*p*<0.05).

**Conclusions:**

MI Varnish TM had a better effect than Clinpro TM White Varnish in remineralizing lesions, in vitro, in the enamel of young permanent teeth with caries in the white-stained phase.

** Key words:**Fluorides varnishes, histological effect, dental remineralization, polarised light microscope.

## Introduction

Fluoride is the most commonly used remineralization agent to combat early caries, as it promotes the formation of fluoride hydroxyapatite (1). In its various forms (gels, varnishes and other fluoride-releasing materials) it can decrease demineralization and increase remineralization of tooth structure (2-4). The major source of topical fluoride comes from toothpastes. However, some studies have found that excessive use of toothpastes can cause dental fluorosis in children (5,6). Therefore, materials have been developed to reduce the risk of fluorosis while promoting remineralization, such as fluoride varnishes (7,8).

Fluoride varnish (FV) is one of the most commonly employed strategies for the management of carious white spot lesions in children, due to its safety and ease of application (9-11). Currently, improved versions of these varnishes by adding components such as the bioactive glass phosphopeptide casein-amorphous calcium phosphate-phosphopeptide (CPP-ACP) and tricalcium phosphate (TCP) enhance their remineralizing action, even at low doses (2,7,8,12).

There are various commercial brands of these versions of FV on the market, with no conclusive evidence on the superiority of any of them, therefore, the objective of the study was to compare the *in vitro* effect of the application of two fluorinated compounds for topical use in the presentation of FV with remineralizers, whose trade names are ClinproTM White Varnish and MI VarnishTM, and its active ingredients are TCP and CPP-ACP, respectively. in the remineralization of the enamel of young permanent teeth with caries in the white spot phase using polarized light microscopy.

## Material and Methods

The study was experimental, *in vitro* and prospective and approved by the Research Ethics Committee of the Faculty of Medicine of the UNMSM (Act 0282). The sample was calculated using G*Power (13) with an α error = 0.05, a power of 80%, and an effect size of 0.8. Based on this, the comparison between two groups using a t-test, indicated the need for 10 tooth fragments per group, consistent with other studies (2,14).

Ten healthy, unfilled permanent premolars were collected from patients aged 12 to 20 years. The premolars were extracted by specialists for orthodontic purposes. The teeth were cleaned with deionised water removing all remaining soft tissue and preserved in sterile polyethylene bottles containing distilled water until processing. They remained in the solution for three months until the experimental procedures.

The sample consisted of 20 tooth fragments (2 fragments of each tooth of 3x5 mm) obtained using a micromotor with plenty of water, a disc and a diamond bur Medmedical Industrial Diamond (MDTR) Made in Israel, Model: 016M. The fragments were induced with carious lesions on the enamel surface by immersing the samples in a demineralizing solution, model proposed by Chokshi *et al*. (14), and placed in a recirculating air oven (Model BOV-V7OF, Temperature RT+10-250°C, Chamber size (mm):400+375+500, Temperature control: 0.1 °C, Voltage: 220V 50Hz, Date of manufacture 2015/03) for 96 hours at 37°C.

Once the white spot lesions were obtained on the tooth fragments, Clinpro TM White Varnish and MI Varnish TM were applied to the 10 fragments, respectively. This process was carried out according to the manufacturer’s instructions. They were then placed in the oven at 37°C for 6 days and each day were subjected to a cyclic PH 3 hours in demineralizing solution and 21 hours in remineralizing solution, simulating the conditions of the oral cavity.

Each fragment was placed in a test tube for fixation, cutting, and reading with NIKON ECLIPSE I series polarized light microscopy with 20X magnification. The depths of the demineralized areas (microns) were measured using Image-Pro plus software and the depth and area of remineralization in each of the groups was calculated.

Statistical analyses were performed using Stata 17.0 statistical software. The Shapiro-Wilk and Levene tests demonstrated normality and homogeneity of variance, respectively, with *p* > 0.05.. Student’s t-test for independent samples was used to evaluate the difference in means with respect to the depth and area of remineralization of the tooth fragments of both groups; while the paired Student’s t-test was used to evaluate the differences within the groups artificially induced white spot lesions. The significance level was evaluated with α = 0.05 and the confidence level was set at 95%.

## Results

According to the paired Student’s t-test,  Table 1 shows the significant differences (*p* ≤ 0.05) between demineralization depth and remineralization depth after application of the BF: Clinpro TM White Varnish (Group I) and MI Varnish TM (Group II).

**Figure 1:**
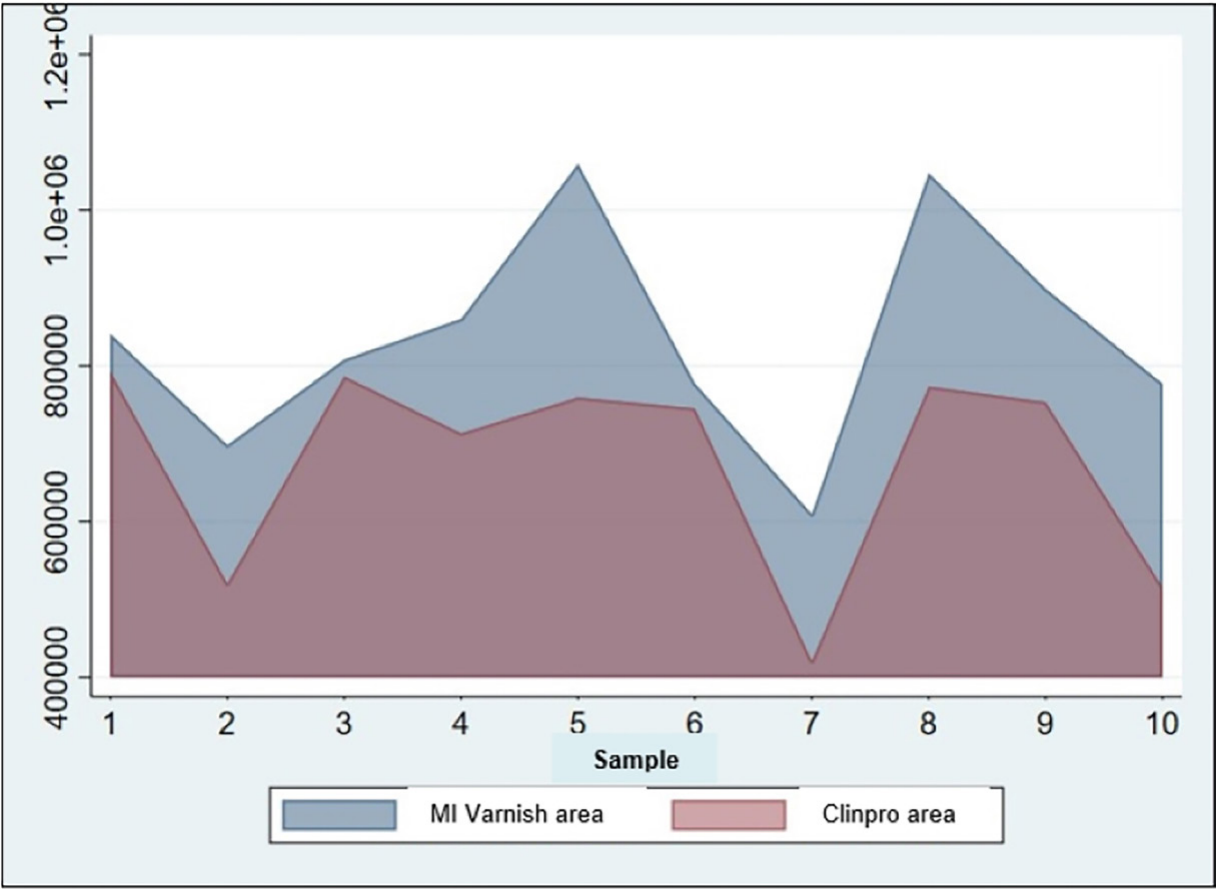
Remineralized areas by group (μm)2.

 Table 2 and Figure 1 show the remineralisation area of the groups, where the MI Varnish TM group achieved a greater remineralisation area than the Clinpro TM White Varnish group. This difference was statistically significant (*p*> 0.05).

**Figure 2:**
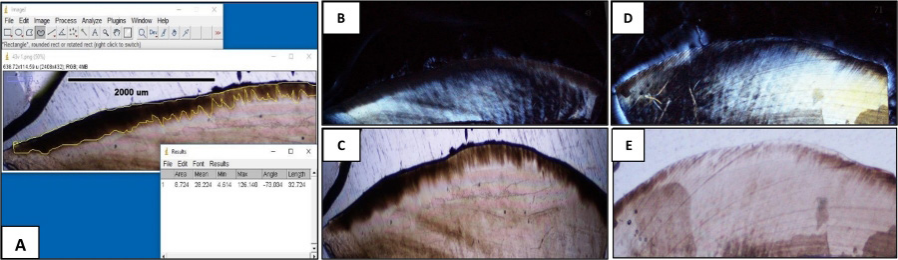
(A-E): Depth measurement with Image Pro Plus software (A) of demineralization ( B and D) and remineralization in enamel with Clinpro TM and Mi varnish TM ( C and E), respectively.

Figure 2 shows the images obtained by polarised light microscopy at demineralization and after remineralization with: MI Varnish TM and Clinpro TM White Varnish. In this sense, favourable changes can be seen after the application of both fluoride agents.

## Discussion

While there are several techniques and methods used to improve demineralization (8,15), few studies have focused on the remineralizing effects of fluoride varnishes with enhancing components such as CPP-ACP and TCP. This study aimed to determine the remineralizing effect of two commercially available BFs, ClinproTM White Varnish (5% sodium fluoride + TCP tricalcium phosphate) and MI Varnish TM (5% sodium fluoride + CPP-ACP Recaldent).

It was found that the depth of demineralization for the samples belonging to the Clinpro TM White Varnish group had a mean of 342.98 μm and the achieved depth of mineralization had a mean of 291.90 μm. While the demineralization depth for those belonging to the MI Varnish TM group had a mean of 306.28 μm and the depth that was mineralized had a mean of 235.74 μm. On the other hand, Clinpro TM White Varnish achieved a mean remineralization area of 678 313.8 μm2, while MI Varnish TM, a mean area of 838 042.6 μm2, a value very similar to that found by Kumar *et al*. (16), whose difference was statistically significant (*p*= 0.0192).

These authors also found that carious lesion depth was lower with MI Varnish TM (226.23±44.25) compared to Clinpro TM White Varnish (285.43±44.70), with no significant differences reported. Similar results to the study by Rani *et al*. (17), also reported higher mean remineralisation areas with the use of MI Varnish TM (92.40±0.09) with statistically significant differences than the other FV (*p*<0.001). It can be concluded that the MI Varnish TM group presented a superior protective potential. The aforementioned could be explained because the CPP-ACP component induces enamel remineralization by enhancing the action of fluoride varnish, as established by Mashhour *et al*. (8).

Other studies have also highlighted the efficacy of CPP-ACP composites. Thakkar *et al*. (18) demonstrated *in vitro* that these compounds are effective in preventing demineralization and promoting remineralization of enamel. Tao *et al*. (19) conducted a systematic review aimed at evaluating the efficacy of the combination of CPP-ACP and fluorides with fluoride monotherapy in patients with early caries lesions. Their results were that fluorides combined with CPP-ACP achieved the same efficacy in smooth surface lesions compared to fluoride monotherapy and that the combination treatment showed better efficacy than fluoride monotherapy for occlusal early caries lesions.

Shaik *et al*. (20) found that after remineralization with two FV, there was a significant difference between the evaluated groups when Ca and P ratios were compared, showing a higher remineralization potential for CPP-ACP followed by Vantej and the Icon group, concluding that the CPP-ACP group performed better in remineralisation of demineralised enamel. Other studies, such as Brar *et al*. (21) have determined that CPP-ACP ( MI Varnish TM ) and TCP (Clinpro TM White Varnish) were excellent delivery vehicles available in a slow-release amorphous form for localising fluoride on the tooth surface, but did not report differences in their remineralization ability.

The superiority of MI Varnish TM in the study may be due to its higher Ca, P and F ion releasing capacity compared to Clinpro TM White Varnish and its longer duration as an active agent (up to 4 weeks) (22). The chemical composition of CPP-ACP contained in MI VarnishTM may also have contributed to its better remineralising performance because it has several properties that aid better remineralization by preventing the growth of ions, keeping them accessible for transport to the regions requiring minerals (17,23), so that ions could diffuse rapidly out of the varnish into the lesions, through the intraprismatic channels (24). In addition, casein had amino acids that acted as a buffer against demineralising agents (16).

Authors such as Salinovic *et al*. (14) found that the mean microhardness values obtained for the group of samples treated with MI Varnish TM were higher compared to the other groups compared (*p* = 0.001), as well as Varma *et al*. (23) found that Mi Varnish TM released more fluoride compared to Clinpro TM White Varnish. However, Ji-Soo *et al*. (25) have established that the superiority of Mi Varnish in releasing fluoride only applies for 12 hours after application, as from 12 to 20 hours after application, Clinpro TM White Varnish released more fluoride. The time was not evaluated in the present study and may be considered in future research.

In contrast to the results of this study and considering that no clear distinction between the two FVs has been reported, Mohd *et al*. (26) evidenced that Clinpro TM White Varnish achieved a higher remineralization than MI Varnish TM (*p*<0.05). Handa *et al*. (27) reported similar results where the success rate of Clinpro TM White Varnish group =67.61%; success rate of MI Varnish TM group =60.59%, although the difference was not statistically significant. Rao *et al*. (28), found that the remineralisation potential of Clinpro TM White Varnish paste was better than Duraphat and ReminPro (*p*<0.05). Also, Rechmann *et al*. (29) when comparing MI Varnish TM and MI Paste Plus, reported no differences in tooth remineralisation. Poza-Pascual *et al*. (30) found no significant effect on pH level, lactic acid concentration, or the amount of elements such as F, Na, Ca, etc. after application of Clinpro TM White Varnish and MI Varnish TM.

Another study indicates a better performance of other substances compared to CPP-ACP composites, such as the study by Chaudhary *et al*. (31) who found that sodium calcium phosphosilicate paste showed a more effective tooth remineralizing potential than CPP-ACP and fluoride toothpastes.

Despite these latter studies, the results of this study indicate that initial caries lesions can be treated in a non-invasive way by remineralisation with compounds containing calcium, phosphates and fluoride. However, this study, being *in vitro*, has certain limitations such as the difficulty in simulating the oral environment, the lower level of proteins and bacteria in the artificial saliva used and the lack of control of the flow rate. Nevertheless, it is recommended to use them for cost/benefit evaluation in public health policies in developing countries.

## Conclusions

FV MI VarnishTM (5% sodium fluoride + CPP-ACP Recaldent) had a better histological effect than FV ClinproTM White Varnish (5% sodium fluoride + tricalcium phosphate TCP) in remineralizing artificially produced lesions(*in vitro*) in the enamel of young permanent teeth with caries in the white spot stage.

## Figures and Tables

**Table 1:** Difference between depth of demineralization and depth of remineralization with two different fluoride varnishes (μm)2.

Group	n	Mean	SD	Minimum	Maximum	t	p
MiVarnish							
Demineralization	10	306.28	88.87	242.70	369.85	2.3646	0.042
Remineralization	10	235.74	48.26	201.22	270.26		
Clinpro							
Demineralization	10	342.98	78.12	287.10	398.87	2.2909	0.0477
Remineralization	10	291.91	70.14	241.73	342.08		

SD = standard deviation; Student’s t-test

**Table 2:** Remineralization treatment area (μm)2 with two different fluoride varnishes.

Group	n	Mean	SD	Minimun	Maximum	t	p
Clinpro	10	678313.8	137265.7	580119.8	776507.8	-2.572	0.0192
MiVarnish	10	838042.6	140359.3	737635.6	938449.6		

SD = standard deviation; Student’s t-test

## Data Availability

The datasets used and/or analyzed during the current study are available from the corresponding author.
